# Impact of the COVID-19 Pandemic on the Prevalence of HAIs and the Use of Antibiotics in an Italian University Hospital

**DOI:** 10.3390/healthcare10091597

**Published:** 2022-08-23

**Authors:** Giovanna Deiana, Antonella Arghittu, Davide Gentili, Marco Dettori, Alessandra Palmieri, Maria Dolores Masia, Antonio Azara, Paolo Castiglia

**Affiliations:** 1 Department of Biomedical Sciences, University of Sassari, 07100 Sassari, Italy; 2 University Hospital of Sassari, 07100 Sassari, Italy; 3 Department of Medicine, Surgery and Pharmacy, University of Sassari, 07100 Sassari, Italy

**Keywords:** healthcare-associated infections, antimicrobial resistance, COVID-19, infection prevention and control

## Abstract

The COVID-19 pandemic has massively affected healthcare systems globally, causing a possible reduction in attention to traditional infection prevention programs. The objective of this study was to estimate the prevalence of healthcare-associated infections (HAIs) and the use of antimicrobials in an Italian University Hospital and to investigate whether the intensification of hospital infection control measures during the COVID-19 pandemic has affected the prevalence of bacterial HAIs. A point prevalence survey was conducted according to the simplified ECDC protocol. The survey identified a local HAI prevalence of 9.0%, revealing an increase compared to pre-pandemic values (7.3%). The survey also identified an antimicrobial exposure of 40.8%, revealing a decrease in their use compared to the study carried out in the pre-pandemic era (44.6%). Among the organizational challenges experienced during the COVID-19 pandemic, despite the greater attention paid to infection prevention measures aimed at reducing SARS-CoV-2, many healthcare facilities had to contend with the controlled availability of personnel, physical space limitations and a large number of patients. Active surveillance in hospital wards and the consequent reporting by personnel specialized in infection control is fundamental for hospitals to recognize gaps in prevention and report any observed increases in HAIs.

## 1. Introduction

Healthcare-associated infections (HAIs) are acquired infections that are the most frequent and serious complication of health care and can occur in any healthcare setting, including acute care hospitals, day-hospital/day-surgery, health care facilities, long-term care clinics, home care, territorial residential structures [1]. The course of many HAIs is further complicated by the emergence of bacterial strains resistant to antibiotics, mainly due to the incorrect or excessive use of these drugs [2,3].

These infections are a major public health concern globally. According to the first global report of the World Health Organization (WHO), HAIs cause a prolonged length of hospital stay, long-term disability, increased resistance of microorganisms to antibiotics, an additional economic burden for health systems and for patients and their families and significant excess mortality [4].

In Europe, HAIs cause 16 million additional days of hospitalization each year, 37,000 attributable deaths and 110,000 deaths, for which infection is a contributing cause. Direct costs alone are estimated at approximately EUR 7 billion. [1]. The magnitude of its effect on society is enormous.

The prevention of HAIs, especially bearing in mind that they affect between 5% and 8% of patients admitted to Italian hospitals, must be considered a specific goal and responsibility for each healthcare professional [5]. Specifically, health surveillance is a fundamental feature of the fight against HAIs, as it enables us to maintain a great level of attention, to define dimensions and characteristics of the problem, to direct interventions, to monitor progress through the use of specific indicators and to promptly identify sentinel events and clusters [6].

Antimicrobial resistance (AMR) continues to be one of the great public health challenges globally [7]. In Europe, estimates based on data from the European Antimicrobial Resistance Surveillance Network (EARS-Net) show that more than 670,000 infections are due to microorganisms resistant to antimicrobials (AMs) and that about 33,000 people die as a direct consequence of these infections [8,9].

The COVID-19 pandemic has massively affected the healthcare systems globally. The widespread transmission of SARS-CoV-2 and increased rates of hospitalization, forcing a reorganization of numerous operating units, resulted in a possible reduction in attention to traditional HAIs prevention programs and to long-established infection control measures [10,11,12]. Several departments are still under pressure due to the constant influx of patients. However, the evidence for the effect of the COVID-19 pandemic on HAIs is still limited and conflicting.

We thus hypothesized that the substantial changes in hospital infection prevention and control (IPC) measures, resulting in particular from the introduction of massive protective measures for healthcare workers (HCWs) during the COVID-19 pandemic, affected the prevalence of bacterial HAIs, thus contributing to the identification of the possible indirect consequences on patients and healthcare systems. Therefore, the primary objective of this study was to estimate the prevalence of HAIs and to assess the use of AMs in an Italian University Hospital. Furthermore, a secondary objective was to compare the results obtained with those recorded in the pre-pandemic period to evaluate the effect of changes in care due to the introduction of anti-COVID-19 protection measures.

## 2. Materials and Methods

### 2.1. Study Setting

The study was conducted by enrolling 41 acute care wards of the University Hospital of Sassari, the main hospital in Sardinia, Italy, in terms of the number and diversity of its technological and professional resources. The hospital, with a percentage of single rooms of 11.5%, comprises 861 beds, two intensive care units (ICU), a long-term care unit (LTC), three internal medicine departments, three general surgery departments, two orthopedic departments and a psychiatric ward. The departments were merged according to the indications of the ECDC protocol. In particular, within the area of medicine were included internal medicine, infectious diseases, hematology, rheumatology, neurology, cardiology, stroke unit, pulmonology, emergency medicine, nephrology and oncology, while in the area of surgery were included general surgery, orthopedics, maxillofacial surgery, pediatric surgery, ophthalmology, cardiac surgery, plastic surgery, vascular surgery, otolaryngology, urology and neurosurgery.

All wards, with the exception of the day-surgery and day-hospital wards, were included in the survey. All patients were included if admitted at 8:00 or earlier and not discharged during the investigation period. Infants were included if born before 8:00 a.m. Patients in the emergency room and patients on dialysis (outpatient) were excluded. The data were collected in a single day for each ward, over a period of four days, from 30 November to 3 December 2021. Patient data were anonymized.

### 2.2. Study Design

A point prevalence survey was conducted according to the simplified ECDC protocol [13]. According to this protocol, patient information was collected only if at least one antimicrobial was prescribed at the time of the visit (with the exception of those prescribed in the 24 h prior to 8:00 on the day of the visit for surgical prophylaxis) or if the patient showed an infection associated with their hospital stay (current or previous). Data on HAIs and/or data on antimicrobials use were collected on a single form for patients with an active care-related infection and/or who received an antibiotic. No information was collected for subjects with no infection and with no antibiotic. For the denominator, all the patients present in the ward were considered.

All kinds of hospital infections were studied according to the diagnostic criteria reported in the ECDC protocol [13]. Diagnosing an infectious disease requires identifying signs and symptoms related to an infection during the investigation or, if signs and symptoms have occurred in the past, the patient is still receiving antibiotics on the day of the investigation. Antimicrobials use often led to the diagnosis of a HAI. For HAIs which had not been treated with antibiotics and treated infections whose characteristics were not consistent with ECDC criteria, the medical records had to be carefully evaluated.

The survey was conducted, using a case-finding algorithm, by doctors from the School of Specialization in Hygiene and Preventive Medicine of the University of Sassari, supported by doctors and nursing referents for the control of infectious risk of each department.

### 2.3. Statistical Analysis

The overall prevalence of HAI related to each ward was calculated as the percentage of infected patients out of the total number of patients observed during each phase of the survey. The prevalence of antimicrobial use was calculated as a percentage of the number of patients who received at least one antimicrobial out of the total number of patients observed.

Categorical data were described using frequency count and percentages. Medians and interquartile ranges were used for continuous variables as they were not normally distributed. The 95% confidence intervals were calculated by applying the exact binomial distribution and setting the past prevalence and AMs exposure values as hypothesized parameters. The data obtained were then compared with those collected in 2019, and differences were tested with the binomial probability test. Statistical significance of *p* < 0.05 was set for all analyses.

The data were collected and analyzed using Excel (Microsoft Office, Microsoft Corporation, Redmond, WA, USA) and the STATA software 16 (StatCorp., Austin, TX, USA).

## 3. Results

A total of 655 patients (76.1% occupancy rate of beds) were included in the study and 61 HAIs were identified in 59 patients (mean age 69.6 ± 14.2; male 72.9%), with an overall prevalence of 9.0% (95% CI 6.9–11.5) and a HAI-patient ratio (No. of HAI/No. of patients with HAI) of 1.03. Prevalence of HAI was higher in the burn center (other) and in the intensive care unit (ICU) (Table 1).

Pneumonia (PN) was the most common HAI, followed by urinary tract infections (UTI) and surgical site infections (SST) (Table 2). Overall, 90.2% of HAIs are attributable to our hospital (6.6% to a different hospital), while the 47.5% of infections can be related to the presence of a device in situ. No HAI was detected in COVID-19 positive patients.

Microbiological investigation was available for 18 HAIs (30.5%) and 20 microorganisms were identified. *Pseudomonas aeruginosa* and *Klebsiella* spp. were the most widespread pathogens, followed by *Escherichia coli*. Furthermore, 16.7% of isolated *Pseudomonas aeruginosa* and 40.0% of *Klebsiella* spp. isolate were resistant to Carbapenems (Table 3). Fortunately, no microorganisms resistant to all drugs were identified.

Overall, AMs exposure was 40.8% (95% CI 37.0–44.6) (Table 4). A total of 351 AMs were administered to 267 patients (mean age 65.4 ± 21.0; male 56.9%). Seventy-two patients (27.0%) received two AMs, while ten patients (3.7%) received three or more AMs. AMs exposure in COVID-19 and non-COVID-19 patients was 18.2% and 41.5%, respectively.

Antimicrobials were administered as treatment in 62.1% of cases. Particularly, the administration of AMs for community acquired infections was 37.3% of the total prescriptions, while the administration for nosocomial infections was 24.8%. Surgical prophylaxis was mainly prescribed for more than one day (12.5%). Single-dose prophylaxis was prescribed in only 2.6% of cases. The prescription for medical prophylaxis was 13.7% (Table 5). A total of 4.8% of AMs was administered with no clear indication.

The most frequently administered AMs were combinations of penicillin and beta-lactamase inhibitors (40.2%), third-generation cephalosporins (14.5%), other antibacterial (7.4%), macrolides (6.3%) and first-generation cephalosporins (4.6%) (Table 6).

## 4. Discussion

The latest national prevalence study, carried out during the pre-pandemic era, found a frequency of patients with an infection contracted during hospitalization equal to 8.0% [5]. In the same period, a previous study performed in our university hospital revealed a prevalence of 7.3% [14]. The present survey identified a local HAI prevalence of 9.0%. This trend was mainly observed, with a statistically significant difference (*p* = 0.000028), in internal medicine wards. On the contrary, in LTC unit we found a significant reduction (*p* = 0.0036). In the previous survey, given the average disease severity, the longer hospitalization and the older age of inpatients, LTC was the ward with the highest prevalence [14]. The relevant improvement obtained is related to the important awareness-raising interventions carried out in this unit (application of the new protocol for the prevention of carbapenemase-producing enterobacteriaceae (CPE), periodic monitoring of the implementation of correct hand hygiene practices, evaluation of the correct isolation of the colonized patient). PN and UTI were the most prevalent HAIs in both the present and the previous study, albeit in reverse order. In the present study, *Pseudomonas aeruginosa* and *Klebsiella* spp. were the most widespread pathogens, while in the previous study, the most frequent were *Klebsiella* spp. and *Escherichia coli* [14].

What has been highlighted can be traced back to the fact that, among the organizational challenges experienced during the COVID-19 pandemic, IPC programs were put to the test, re-proposing hospital hygiene issues that had been partially forgotten in recent decades [15,16,17]. Although greater attention to infection prevention measures aimed at reducing SARS-CoV-2 spread could have led to a diminution in the transmission of HAIs, many healthcare facilities had to contend with the controlled availability of personnel, physical space limitations and a large number of patients [18,19]. Our hospital system faced the COVID-19 pandemic with an additional 100 beds and more than 2500 COVID-19 patients. As a matter of fact, numerous risky behaviors were in place.

Personal protective equipment (PPE), given the fear of being infected, were primarily utilized more for the personal protection of healthcare workers from COVID-19 than for patient protection from HAIs, thereby reducing compliance with IPC measures and increasing the risk of cross-contamination [20,21]. In parallel, the potential circulation of CPE may also have been exacerbated by an increased percentage of antimicrobial prescription in the lack of clear guidelines [22,23].

Moreover, the practice of giving priority for isolation rooms to quarantine COVID-19 affected patients and accommodating them together in dedicated wards without the possibility of containing patients colonized with CPE might have led to the introduction of colonized patients, followed by possible propagation and hospital transmission of the CPE [22,24]. It is also important to point out that, during the pandemic, many hospitals limited or inhibited visits, meaning that HAIs were almost entirely caused by patient-to-patient or HCW-to-patient transmission [25,26,27].

The survey also identified an AM exposure of 40.8%, revealing a decrease in their use compared to the study carried out in the pre-pandemic era (44.6%) [14]. In this case, the reduced use of antibiotics can be linked to the variety of measures adopted before and during the pandemic by our University Hospital in terms of raising awareness and regulating the correct use of antibiotics. In both studies AMs were administered mainly as treatment and the most frequently administered AMs were combinations of penicillins and beta-lactamase inhibitors [14].

The IPC measures are fundamental interventions to reduce the impact of HAIs and, in general, to reduce the spread of antibiotic-resistant microorganisms. Not all HAIs are preventable, but it is currently estimated that more than 50% may be [28,29]. One of the crucial aspects in the fight against HAIs is the definition and application of good care practices according to integrated programs that must be adapted to each care setting, including the establishment, in each hospital, of a multidisciplinary committee, an operational group and dedicated nursing staff [30].

It has been empirically shown that participation in active surveillance systems for HAIs is associated, over time, with a reduction in the incidence of infections [31,32,33,34,35]. Several countries in Europe have already introduced effective action strategies to control the HAI phenomenon, although Italy still lacks a national governance system that includes the notification and standardization of HAI surveillance.

However, evidence on the effect of COVID-19 on HAIs is still limited. On one side, some data, such as those from our study, suggest an important impact of the COVID-19 pandemic on HAIs [22,36]. On the other side, recent studies have observed a positive indirect role of the IPC measures, adopted to contain SARS-CoV-2 transmission, on HAI prevention [37,38]. As a matter of fact, additional investigation is necessary to quantify the influence that all these factors may have had on HAI onset.

This study presents several strengths and limitations. Its main strength, being based on a validated ECDC methodology, is its great comparability over time. If used regularly, it could help to calculate temporal trends and epidemiological variations following the implementation of IPC strategies. As for its possible limitations, being a single-center study, it would be difficult to generalize the results to other centers, which may have different IPC procedures.

## 5. Conclusions

Our data may give an interesting contribution to the research in this field, as they describe how patient safety, in particular HAI prevalence, might have been affected by the COVID-19 pandemic. Many hospitals had to face extraordinary conditions of increased patient workload, unprecedented staffing challenges, and numerous operational changes that limited the application and effectiveness of standard infection prevention practices, highlighting how IPC strategies require considerable efforts at the operational, administrative, organizational and, above all, national governance levels.

The present study highlights how active surveillance in hospital wards and the consequent reporting by personnel specialized in infection control is fundamental to recognize gaps in prevention and report any observed increases in HAIs. Surveillance, especially a long-term prospective one, increases awareness among HCWs, consequently reducing HAI incidence [39,40,41]. Obviously, the quality of surveillance is essential as higher surveillance quality provides higher prevalence of HAIs. The importance of surveillance and research is also underlined by the global action plan for antimicrobial resistance, according to which it is essential to create and apply specific protocols and guidelines.

Infection prevention teams should continue to strengthen infection prevention practices and consider the significance of building resilience in their programs to endure future public health emergencies. Indeed, hospital spaces, paths and facilities need to be rethought to be more effective in combating HAIs while promoting the diffusion of good practices among healthcare personnel.

## Figures and Tables

**Table 1:** Prevalence of healthcare-associated infections by ward specialty.

Areas	Patients	Patients with at Least One HAI	Prevalence (%) of HAIs (95% CI)
Geriatrics	32	4	12.5 (3.5–29.0)
Obstetrics/Gynecology	48	1	2.1 (0.1–11.1)
Medicine	272	35	12.9 (9.1–17.4)
Neonatology	23	0	0.0
Pediatrics	12	0	0.0
Psychiatry	9	0	0.0
Surgery	196	10	5.1 (2.5–9.2)
Other	3	1	33.3 (0.8–90.1)
Intensive Care Unit	34	7	20.6 (8.7–37.9)
Long-Term Care	26	1	3.9 (0.1–19.6)
**Total**	**655**	**59**	**9.0 (6.9–11.5)**

**Table 2:** Pathogens causing healthcare-associated infections.

Pathogens	HAI Type, No. and %													
	PN 20 (32.8)	UTI 11 (18.0)	SST 6 (9.8)	GI 4 (6.6)	SSI 4 (6.6)	BSI 3 (4.9)	CRI 3 (4.9)	SYS 3 (4.9)	CVS 2 (3.3)	LRI 2 (3.3)	BJ 1 (1.6)	NOS 1 (1.6)	REPR 1 (1.6)	Total 61 (100.0)
No. (%) of infections (at least one pathogen isolated)	2 (10.0)	9 (81.8)	0 (0.0)	0 (0.0)	0 (0.0)	1 (33.3)	2 (66.7)	1 (33.3)	0 (0.0)	1 (50.0)	0 (0.0)	1 (100.0)	1 (100.0)	18 (29.5)
Total isolated pathogens	2	11	0	0	0	2	2	1	0	1	0	1	1	21
**Gram-negative bacteria**	**1 (50.0)**	**9 (81.8)**	**0**	**0**	**0**	**1 (50.0)**	**1 (50.0)**	**1 (100.0)**	**0**	**1 (100.0)**	**0**	**1 (100.0)**	**0**	**15 (71.4)**
*Escherichia coli*	0	2 (18.2)	0	0	0	0	0	1 (100.0)	0	0	0	0	0	3 (14.3)
*Klebsiella pneumoniae*	1 (50.0)	3 (27.3)	0	0	0	0	0	0	0	0	0	1 (100.0)	0	5 (23.8)
Other *Klebsiella* spp.	0	1 (9.1)	0	0	0	0	0	0	0	0	0	0	0	1 (4.8)
*Pseudomonas aeruginosa*	0	3 (27.3)	0	0	0	1 (50.0)	1 (50.0)	0	0	1 (100.0)	0	0	0	6 (28.6)
**Gram-positive bacteria**	**1 (50.0)**	**2 (18.2)**	**0**	**0**	**0**	**1 (50.0)**	**1 (50.0)**	**0**	**0**	**0**	**0**	**0**	**0**	**5 (23.8)**
*Enterococcus faecalis*	0	1 (9.1)	0	0	0	0	0	0	0	0	0	0	0	1 (4.8)
Other *Enterococcus* spp.	0	1 (9.1)	0	0	0	0	0	0	0	0	0	0	0	1 (4.8)
*Staphylococcus aureus*	1 (50.0)	0	0	0	0	1 (50.0)	0	0	0	0	0	0	0	2 (9.5)
*Staphylococcus epidermidis*	0	0	0	0	0	0	1 (50.0)	0	0	0	0	0	0	1 (4.8)
**Fungi**	**0**	**0**	**0**	**0**	**0**	**0**	**0**	**0**	**0**	**0**	**0**	**0**	**1 (100.0)**	**1 (4.8)**
*Candida albicans*	0	0	0	0	0	0	0	0	0	0	0	0	1 (100.0)	1 (4.8)

PN—pneumonia; UTI—urinary tract infection; SST—skin and soft tissue infection; GI—gastrointestinal system infections; SSI—surgical site infection; BSI—bloodstream infection; CRI—catheter-related infection; SYS—systemic infection; CVS—cardiovascular system infection; LRI—lower respiratory tract infection; BJ—bone and joint infection; NOS—not specified; REPR—reproductive tract infection.

**Table 3:** Antimicrobial resistance of tested microorganisms.

Pathogens	No.	Oxacillin		Glycopeptides		Third-Generation Cephalosporins		Carbapenems	
		No (%) of Tested	No (%) of Resistance	No (%) of Tested	No (%) of Resistance	No (%) of Tested	No (%) of Resistance	No (%) of Tested	No (%) of Resistance
*Escherichia coli*	3	-	-	-	-	2	1 (50.0)	3	1 (33.3)
*Klebsiella* spp.	6	-	-	-	-	6	2 (33.3)	5	2 (40.0)
*Staphylococcus* spp.	3	3	1 (33.3)	3	0 (0.0)	2	0 (0.0)	-	-
*Enterococcus* spp.	2	-	-	2	0 (0.0)	2	1 (50.0)	-	-
*Pseudomonas aeruginosa*	6	-	-	-	-	-	-	6	1 (16.7)

**Table 4:** Prevalence of antimicrobials use by ward specialty.

Areas	Patients	Patients with at Least One Prescribed AMs	Prevalence of AMs Exposure (95% CI)
Geriatrics	32	20	62.5 (43.7–78.9)
Obstetrics/Gynecology	48	7	14.6 (6.1–27.8)
Medicine	272	123	45.2 (39.2–51.3)
Neonatology	23	4	17.4 (5.0–38.8)
Pediatrics	12	5	41.7 (15.2–72.3)
Psychiatry	9	0	0.0
Surgery	196	71	36.2 (29.5–43.4)
Other	3	2	66.7 (9.4–99.2)
Intensive Care Unit	34	21	61.8 (43.6–77.8)
Long-Term Care	26	14	53.9 (33.4–73.4)
**Total**	**655**	**267**	**40.8 (37.0–44.6)**

**Table 5:** Antimicrobials prescribed according to the indications for use.

Indications	No. of Antibiotics	% of Total Prescriptions
**Therapy**	**218**	**62.1**
Treatment of community-acquired infection	131	37.3
Treatment of hospital-acquired infection	87	24.8
**Prophylaxis**	**110**	**31.4**
Medical prophylaxis	48	13.7
Surgical prophylaxis: single dose	9	2.6
Surgical prophylaxis: one day	9	2.6
Surgical prophylaxis: >1 day	44	12.5
**Other indications/unknow**	**23**	**6.5**
Other reason	6	1.7
Unknown indication	17	4.8
**Total**	**351**	**100.0**

**Table 6:** Antimicrobials prescribed according to the pharmacological class.

Antibiotics	No. of Antibiotics	% of Total Prescriptions
Combinations of penicillins, including beta-lactamase inhibitors	141	40.2
Third-generation cephalosporins	51	14.5
Other antibacterials	26	7.4
Macrolides	22	6.3
First-generation cephalosporins	16	4.6
Carbapenems	13	3.7
Fluoroquinolones	12	3.4
Other aminoglycosides	12	3.4
Glycopeptide antibacterials	11	3.1
Triazole derivatives	7	2.0
Imidazole derivatives	7	2.0
Other antimycotics for systemic use	6	1.7
Tetracyclines	6	1.7
Penicillins with extended spectrum	5	1.4
Combinations of sulfonamides and trimethoprim, incl. derivatives	4	1.1
Lincosamides	4	1.1
Antibiotics (Intestinal Antiinfectives)	3	0.9
Antibiotics (Drugs for treatment of tuberculosis)	2	0.6
Fourth-generation cephalosporins	2	0.6
Beta-lactamase-resistant penicillins	1	0.3
**Total**	**351**	**100.0**

## Data Availability

The data presented in this study are available on reasonable request from the corresponding author.
